# Participatory design of an improvement intervention for the primary care management of possible sepsis using the Functional Resonance Analysis Method

**DOI:** 10.1186/s12916-018-1164-x

**Published:** 2018-10-11

**Authors:** Duncan McNab, John Freestone, Chris Black, Andrew Carson-Stevens, Paul Bowie

**Affiliations:** 1 NHS Education for Scotland, 2 Central Quay, Glasgow, Scotland G3 8BW UK; 2 NHS Ayrshire and Arran, Ayr, UK; 3 Institute of Health and Wellbeing, University of Glasgow, Glasgow, UK; 4 Division of Population Medicine, School of Medicine, Cardiff University, Cardiff, UK; 5 Department of Family Practice, University of British Columbia, Vancouver, Canada; 6 Australian Institute of Health Innovation, Macquarie University, Sydney, Australia

**Keywords:** Quality improvement, Complexity, Functional resonance analysis method, Sepsis, Primary care

## Abstract

**Background:**

Ensuring effective identification and management of sepsis is a healthcare priority in many countries. Recommendations for sepsis management in primary care have been produced, but in complex healthcare systems, an in-depth understanding of current system interactions and functioning is often essential before improvement interventions can be successfully designed and implemented. A structured participatory design approach to model a primary care system was employed to hypothesise gaps between work as intended and work delivered to inform improvement and implementation priorities for sepsis management.

**Methods:**

In a Scottish regional health authority, multiple stakeholders were interviewed and the records of patients admitted from primary care to hospital with possible sepsis analysed. This identified the key work functions required to manage these patients successfully, the influence of system conditions (such as resource availability) and the resulting variability of function output. This information was used to model the system using the Functional Resonance Analysis Method (FRAM). The multiple stakeholder interviews also explored perspectives on system improvement needs which were subsequently themed. The FRAM model directed an expert group to reconcile improvement suggestions with current work systems and design an intervention to improve clinical management of sepsis.

**Results:**

Fourteen key system functions were identified, and a FRAM model was created. Variability was found in the output of all functions. The overall system purpose and improvement priorities were agreed. Improvement interventions were reconciled with the FRAM model of current work to understand how best to implement change, and a multi-component improvement intervention was designed.

**Conclusions:**

Traditional improvement approaches often focus on individual performance or a specific care process, rather than seeking to understand and improve overall performance in a complex system. The construction of the FRAM model facilitated an understanding of the complexity of interactions within the current system, how system conditions influence everyday sepsis management and how proposed interventions would work within the context of the current system. This directed the design of a multi-component improvement intervention that organisations could locally adapt and implement with the aim of improving overall system functioning and performance to improve sepsis management.

**Electronic supplementary material:**

The online version of this article (10.1186/s12916-018-1164-x) contains supplementary material, which is available to authorized users.

## Background

Sepsis is a life-threatening condition where tissue damage, organ failure and death may result due to the body’s own response to infection [1, 2]. It is thought to cause at least six million deaths per annum worldwide, many of which are thought to be preventable with early recognition and treatment [1, 2]. There is international expert consensus that increased awareness, earlier presentation and detection, rapid administration of antibiotics and treatment according to locally developed guidelines can significantly reduce sepsis-related deaths [3, 4]. In secondary care, compliance with care protocols for patients with signs suggestive of sepsis is believed critical to improving outcomes and minimising sepsis-related deaths [5]. However, the implementation of sepsis management interventions has been problematic with only 10–20% of patients receiving care that is fully compliant with intervention recommendations [6, 7].

While a significant amount has been reported about work undertaken within the hospital setting to improve sepsis management, work in primary care is at a much earlier stage but has become a national priority in Scotland [8–11]. Presentations with infective conditions in this setting are exceedingly common, with only a very small proportion developing sepsis, while initial symptoms of sepsis can be vague—making early, accurate identification of patients who have sepsis or may develop it a challenge [12]. In several high-profile cases, primary care management of patients who had sepsis was thought to be inadequate [13, 14]. Guidelines to aid the identification of acutely ill patients who may have sepsis in primary care have been published that recommend the use of a structured set of clinical observations to stratify the risk of sepsis including pulse, temperature, blood pressure, respiratory rate, peripheral oxygen saturation and consciousness level [10].

Quality improvement (QI) as both a philosophy and suite of methods [15] has underpinned the design of major national preventive efforts to tackle sepsis internationally [16–18]. Recent perspectives on QI argue that in complex healthcare systems the design of improvement interventions risks being flawed if there is limited focus beforehand to gain a deep insight into how the system under study actually functions when things go right and wrong [19–26].

Primary healthcare has been described as a complex socio-technical system [28, 30]. Such systems consist of many dynamic and interacting components (e.g. clinicians, patients, tasks, information technology, protocols, equipment and culture) and are affected by rapid changes in conditions (such as patient deterioration, reduced staff capacity, increased patient demand, limited information and availability of resources) [28–31]. Often, different parts of systems can be closely coupled resulting in changes in one area affecting other areas in a non-linear, unpredictable manner. Rather than being purposively designed, systems of work often emerge and evolve over time due to the interactions between different components. People employ workarounds (for example, when information is not available) and trade-offs (such as when staff have to prioritise task efficiency over thoroughness) to achieve safe care [31–34]. “Work-as-done” (WAD), including performance adjustments, represents everyday work and is often different from “work-as-imagined” (WAI) as encapsulated in clinical guidelines and protocols and imagined by those in other parts of the system such as senior managers and policymakers.

Healthcare improvement projects to implement recommendations or clinical guidelines are often complex interventions that include multiple interacting and interdependent components; for example, education, new care protocols, new staff roles and new ways of accessing services [19, 20]. There is a growing awareness of the importance of understanding the complexity of current work and considering interactions between proposed interventions and the existing system in the planning and design stages of improvement projects to inform potential success [24–26].

The rationale for this study was to explore and better understand how acutely ill patients who may have sepsis are currently identified and managed in the community, obtain multiple perspectives on potential improvement interventions and determine how best these suggestions can inform the design of a system-centred improvement intervention.

## Methods

The methods and results of this project have been reported in keeping with current, best practice guidelines advised by Tong et al. [35]. A COREQ checklist (Additional file 1) is included as Table 6 in Appendix 1.

### Clinical setting

The study was conducted in a primary care setting within a single, Scottish, regional health board, NHS (National Health Service) Ayrshire and Arran (NHSAA). The identification and management of sepsis is a priority patient safety improvement focus for NHSAA but the best way to design and implement a related intervention in community settings was not clear to local clinical leaders, management and improvement advisors. To access appropriate treatment including antibiotics and fluid management, patients may self-present at the hospital Emergency Department (ED) either by themselves or through telephoning for an ambulance. Alternatively, they may be assessed in the community by a general practitioner (GP) or advanced nurse practitioner (ANP). During normal working hours (8:00 am to 6:00 pm Monday to Friday), clinical assessment is arranged by GP reception staff, while at other times it is arranged by NHS24 (a special national health board within NHS Scotland that provides health information and facilitates patient access to primary care out-of-hours services provided regionally by Ayrshire Doctors On Call (ADOC)). Other healthcare professionals, such as nurses who work in the community and in nursing care homes, can arrange out-of-hours clinical review directly using the single point of contact (SPOC—a non-clinical administrative member of staff who arranges ADOC appointments directly based on the instruction from the healthcare professionals). If, after clinical assessment, it is thought that admission is required, clinicians discuss secondary care assessment with colleagues in the Combined Medical Assessment Unit (CMAU) and then forward documentation summarising their findings and presumed diagnosis and arrange transport.

### Study design

A mixed methods approach, including semi-structured interviews, group interviews and documentary analysis, was used to identify system functions and their interactions and output variability to inform a contextually grounded design of a Functional Resonance Analysis Method (FRAM) model [36, 37]. Multiple clinical, management and administrative perspectives on potential system improvements were identified and themed. A participatory design approach [38] using a key stakeholder workshop was then used to reflect on FRAM findings and improvement suggestions and identify and agree improvement interventions based on a systems approach to this issue.

### Functional Resonance Analysis Method (FRAM)

The Functional Resonance Analysis Method (FRAM) is one way to begin to model and understand non-trivial, complex, socio-technical systems [36]. The FRAM involves exploring “work-as-done” with frontline workers to identify the “functions” that are being performed. A function is defined as “the activities—or set of activities—that are required to produce a certain outcome” [36]. Identified system functions are entered into the FRAM Model Visualiser software (FMV). FRAM studies the relationships within a system by exploring potential interactions between functions to identify coupling between different parts of the system. To achieve this, links are created between functions by identifying six specific aspects of each function: input, output, preconditions, resources, controls and time factors (Table 1). For example, the output of a function <book appointment> is <appointment booked> which is a precondition of the function <perform clinical assessment>. A key component of the FRAM is to study and record the variability of the output of each function. Functional resonance refers to how variability of different functions can combine to produce amplified and unpredicted effects (both wanted and unwanted).

**Table 1:** Aspects of FRAM functions

Aspect	Description	Example for function <perform clinical assessment>
Input (I)	What the function acts on or changes and starts the function	Patient arriving at the consulting room
Output (O)	What emerges from the function—this can be an outcome or a state change	Clinical assessment complete
Precondition (P)	Some condition that must be met before the function can start	Appointment booked
Resources (R)	Anything (people, information, materials) needed to carry out the function or anything that is used up by the function	Thermometer, stethoscope
Control (C)	Anything that controls or monitors the function	Protocol or guidelines
Time (T)	Time constraint that may influence the function	10-min consultation

The FRAM is one method to facilitate the adoption of a complex systems approach. Exploring and building a model of work-as-done allows consideration of how people adapt to deal with unexpected clinical presentations, system conditions (such as availability of information or time) and competing goals (such as efficiency and thoroughness). Exploring how these adaptations combine with variability elsewhere in the system encourages a shift from considering systems as linear, where event A causes outcome B in a predictable manner, to adopting a complex systems approach to focus on the relationships between components and how outcomes emerge from these interactions. FRAM has previously been used in healthcare to explore the complexity of the system for taking blood prior to blood transfusion [39] and to guide implementation of guidelines [40] by exploring current work systems with health care professionals to ensure proposed changes were compatible with current ways of working. It is used regularly in parts of Denmark to explore complex systems in order to plan improvements [41].

Real linkages can only be found by looking at the system with a specified set of conditions, such as an event that has occurred or by predicting how a particular event may occur—these are called instantiations. The linkages present in any given instantiation are a subset of all the potential linkages in the FRAM model and can be used to understand how historical events occurred, consider how the system may perform in varying conditions or how system performance may be altered by change to one function. The FRAM also describes variability of function output. This variability, or functional resonance, reflects the normal, everyday variability of function output caused by altering system conditions and the adaptations people employ to continue successful operations in these conditions. Rather than being quantified, variability is recorded as present or not within a function and can be described as too early, on time, too late, not at all, precise, acceptable and imprecise. Resonance (or variability) in one function can combine with resonance in other functions and lead to unpredicted outcomes both positive and negative.

### Study participants

A pragmatic, purposive sampling strategy was employed to identify appropriate healthcare professionals working in primary, secondary and interface care settings with experience and knowledge of their part of the NHSAA Sepsis identification and management system who were then invited to participate in semi-structured interviews. Twenty-two healthcare professionals and administrators were contacted by email and all agreed to participate. Fifteen interviews were completed (Table 2).

**Table 2:** List of interviews

Professional role	Number of interviewees	Individual or group interview
General practitioners with both in-hours and out-of-hours roles	4	Individual
GP specialty trainee—who work both in and out-of-hours	1	Individual
In-hours ANPs	2	Group
Out-of-hours advanced nurse practitioners	1	Individual
NHS 24 nursing staff	5	Group
ADOC administrative staff (single point of contact and reception staff)	2	Individual
Combined assessment unit (secondary care) senior nurse	1	Individual
Accident and emergency senior nurse	1	Individual
Accident and emergency consultant	1	Individual
General practice receptionist	2	Group
Community nurses	2	Group

To assess variability of functions, ADOC were asked to provide relevant out-of-hours data and a pragmatic, convenience sample of NHSAA general practices was approached to provide in-hours data (Table 3). Twenty (of 55 NHSAA) general practices were asked to provide data on recent admissions of which eight practices returned requested data (40%).

**Table 3:** Data extracted from ADOC electronic records

Date and time seen Age Case summary (consultation text and values) Diagnostic codes applied Priority assigned by NHS24 (to be seen within 1, 2 or 4 h) The use of a specific sepsis template (yes/no)	

### Data collection and analysis

The following data collection, interpretation and analytical methods were applied to enable construction of a preliminary FRAM Model, identify and theme improvement suggestions and design an improvement intervention.

#### Semi-structured interviews

Fifteen semi-structured, face-to-face, individual (*n* = 11) and group (*n* = 4) interviews were conducted at the participants’ place of work by DM. Only DM, who is a GP in the area and an experienced qualitative researcher, and the participants were present during interviews, and no repeat interviews were conducted. The duration of interviews was from 22 to 54 min. Study aims were explained and a definition of sepsis was provided to participants. Interviews were informed by an inductive approach [42] and structured in design to ensure data collection identified functions and their aspects to construct the FRAM model and suggestions for system improvement.

#### GP in-hours data

Participating GP practices (*n* = 8) provided data on their last ten admissions for adults with a presumed infective cause (chest infection, urine infection, cellulitis or other presumed infective cause based on the recorded consultation). A worksheet was completed by either a GP within the practice or the practice manager to record if the following were explicitly stated in the admission letter: patient’s pulse, temperature, oxygen saturations, blood pressure, a comment on level of consciousness and if a working diagnosis of sepsis or possible sepsis was noted.

#### GP out-of-hours data

Anonymised data for all acute hospital admissions was extracted from the ADOC computer system for a full calendar month in 2016 and downloaded to MS Excel Software [Microsoft Corporation, version 12.0 / 2007] for analysis (Table 3). Patients aged 16 or over admitted with a suspected infective cause were identified and selected by the lead author (DM). The Microsoft Excel random number generator was used to select 50 patient cases, which the research team agreed should be sufficient to provide evidence of variability within this part of the system.

#### Identification of system functions and aspects

All individual and group interviews with participants were audio-recorded and transcribed with consent. A systematic and iterative approach to analysis of the interview data based on the constant comparative method was adopted [43]. Transcription text was read and re-read by DM to facilitate a deep understanding of the data. Functions required in the current system for the identification and management of sepsis were identified and treated as themes. Responses were coded within QDA Miner [Provalis Research, Montreal, Canada, Version 1.4.6.0, 2002] based on these themes. The data for each theme was analysed to identify aspects of each function. All data were cross checked with other authors with any disagreements resolved by discussion until consensus was achieved. Finally, system functions and aspects were uploaded to FMV software [Zerprize, New Zealand, Version 0.4.1, 2016].

#### Assessment of variability of function output

Variability of function output was assessed through analysis of interview data for reported variability in function output. In addition, out-of-hours and in-hours admission data was analysed to determine the number and percentage of patients with each physiological parameter recorded, the number and percentage with all parameters recorded and the median number of physiological parameters recorded per patient. The median was calculated as it was thought that some practices may have either very high or very low levels of recording physiological parameters [44]. For out-of-hours admissions, the use of an electronic template for recording observations and priority (1, 2 or 4 h) assigned by NHS24 was recorded. This was determined for all patients and separately for those with a presumed diagnosis of sepsis. Variability of function output was entered into the FMV software.

#### Design of improvement intervention

A separate thematic analysis identified suggested areas for system improvement. Suggestions from interviewees were coded in QDA Miner by DM and arranged into themes through discussion of codes by authors (DM, JF and CB). A workshop was held for key local stakeholders with primary care management, leadership and frontline clinical roles (*n* = 6) to both validate the FRAM model and gain consensus on improvement priorities and strategies. Through discussion, the FRAM model was used to reconcile improvement suggestions with work-as-done and consensus was sought on the design of an improvement intervention. A Driver Diagram was constructed to link the overall aim of the project with the major improvement drivers identified enabling a multi-component improvement intervention strategy to be designed [45]. Consensus was deemed to have been reached when full agreement was achieved by all attendees.

## Results

### FRAM model

**Fig. 1:**
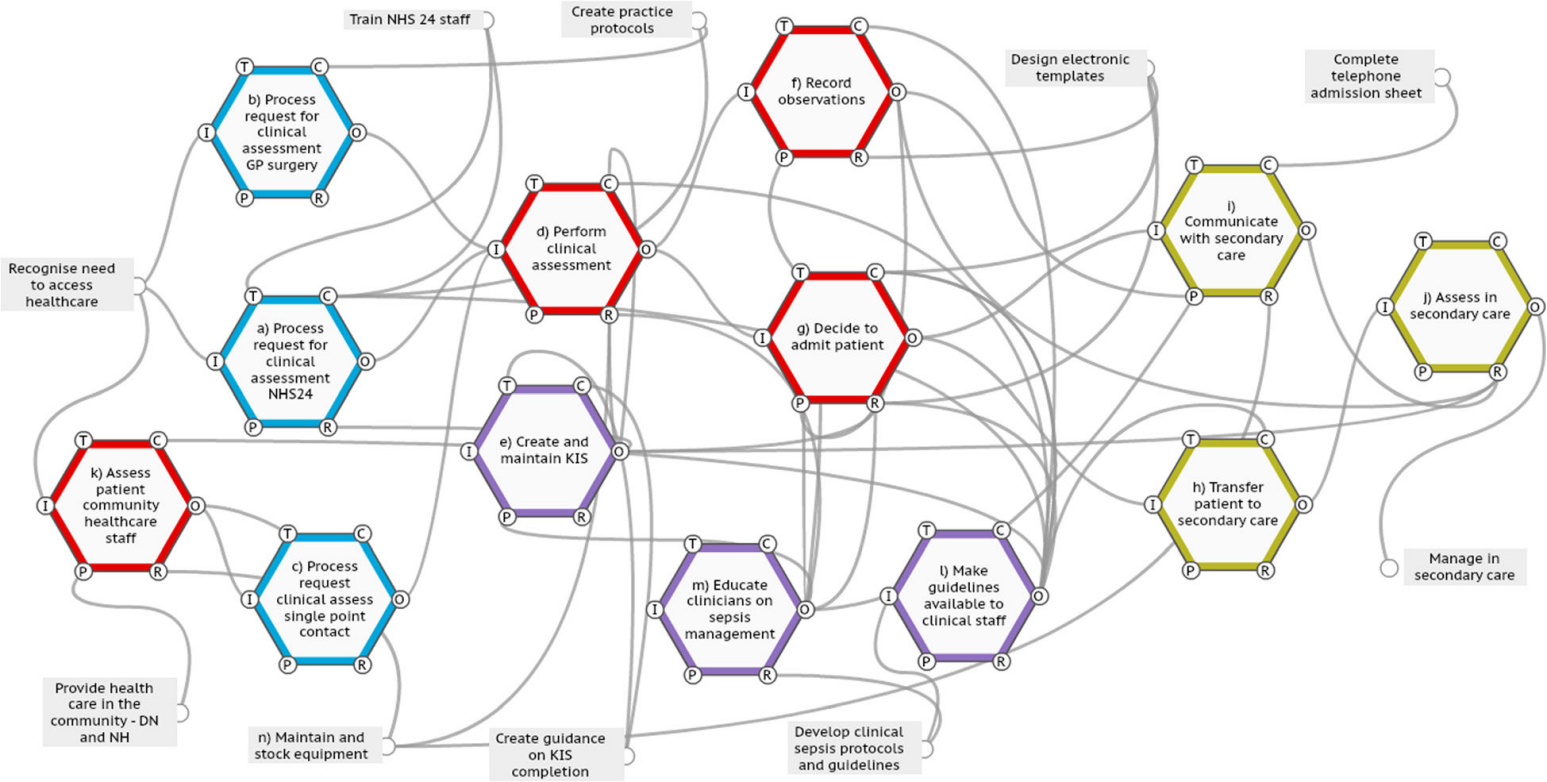
Functional Resonance Analysis Method (FRAM) model of system to identify and clinically manage sepsis in primary care in NHSAA

Fourteen foreground system functions were identified with description of the function and output variability outlined in Tables 4 and 5 (Fig. 1). Seventeen background functions were required to complete the FRAM model of which the key stakeholder group felt ten were relevant to discussions on improvement intervention design. For example, the function <Create guidance on KIS completion> was not the focus of the FRAM; therefore, its aspects were not explored, meaning it only had an output and was thus a background function. It was considered relevant in the design of the improvement intervention as change to this function may influence the function <Create and maintain KIS>. In contrast, it was thought that an intervention would be unlikely to influence the background function <Manage staff capacity> and so this was not included in the FRAM model that was discussed.

**Table 4:** Functions from the Functional Resonance Analysis Method (FRAM) model

Function	Description of influence of system conditions on function and output variability Data from audit in bold Quotes from interviews in italics
a) Process request for clinical assessment NHS24	• Capacity/demand mismatches (more requests from patients to speak to staff than number of staff available to meet this demand) may delay commencement of this function. • Staff reported deviating from the algorithm (which may be considered a control) when necessary in an attempt to achieve success *We have got the algorithm but quickly you learn that it's only a guide. I mean, when I was new I used to stick to it but now I do not refer to it. I mean I know it in my head anyway, but I ask other things and get them to hold the phone next to them to hear the breathing, ask them if they feel warm and ask them about confusion. I think that is more helpful.* NHS24 • Variability of assigned triage times was observed with no association between triage time and the likelihood of a patient subsequently being admitted with suspected sepsis. **NHS24 triage time,** ***n*** **(%), when admitted with infective cause from out-of-hours** **○ 1 h = 12 (24)** **○ 2 h = 18 (36)** **○ 4 h = 20 (40)** **NHS24 triage time,** ***n*** **(%), when sepsis suspected at out of hours** **○ 1 h = 7 (24)** **○ 2 h = 10 (34)** **○ 4 h = 12 (41)**
b) Process request for clinical assessment GP surgery	• There was a difference between work-as-done by administrative staff and work-as-imagined by the GPs. **○** *In general, our staff are good at saying this person doesn’t sound well and they are concerned and they don’t call often and they let us know so they will put it onto the emergency doctor.* GP3 **○** *I don’t know if I would necessarily recognise it in a patient coming in because a lot of it is like fever and sickness - it could be anything. Training or a checklist may help.* Receptionist 2 **○** *I think it is easy for us to recognise someone that comes in with chest pains rather than someone who comes in with sepsis.* Receptionist 1 • Capacity/demand issues influenced function output resulting in staff taking less time to assess potential urgency of the medical condition at busy periods. **○** *It can be quite hard on a Monday morning when you have got lots of patients waiting for an on-the-day appointment and we just get a sea of people it would be quite hard to say then could you give me indication of the problem.* Receptionist 2 • Resources such as training, experience and knowledge of the patient were also thought to influence function output. • There were no guidelines or protocols in place for staff. These may act as potentially beneficial controls that help staff to decide actions such as the urgency of speaking to a GP—when to interrupt and when to wait. **○** *I think it’s difficult I don’t think they have had adequate training on it I don’t think years of practice or as a Health Board have addressed training for admin reception type staff.* GP3
c) Process request for clinical assessment by an out-of-hours clinician via the single point of contact	• Output was based on the information given by community healthcare workers and was thought to be variable. • There was no guidance to direct the required urgency of clinical assessment.
d) Perform clinical assessment	• Resource availability to aid clinical assessment was thought to be adequate in both in-hours and out-of-hours care. • In-hours electronic templates were thought to be more useful. **○** *In the surgery we have a template we use that is easy and helpful.* GPANP **○** *The out-of-hours template makes it more difficult – you see it when you are back in the car writing up the case after you have made your decision – it’s too late. I think if it was quick, easy and straightforward you might get better recording (of observations).* GP2 • Clinicians stated that patients with possible sepsis would take more time to assess and manage. This was not thought to influence actions with these patients, but would cause increased time pressure when consulting with subsequent patients. **○** *Time is a major factor although when you are dealing it is not a factor because you blank everything else out and you deal with it - you have to suck it up after.* GPANP **○** *I think often these patients are unwell so you take the time anyway.* GPANP • It was felt that the lack of information available through the Key Information Summary (KIS), an electronic summary of important clinical and social information created by the patient’s GP practice and available to out-of-hours GPs and secondary care, could influence clinical assessment as usual physiological parameters were not available
e) Create and maintain KIS	• The information contained in KIS was noted to be variable by GPs and by hospital teams. This was thought to reflect both a lack of guidance on completion and lack of time to perform this task properly by in-hours clinical teams. **○** *I think it is variable sometimes it is excellent (the KIS) and it makes such a difference - and then other times it is not - and I think that is probably one of the reasons why it is not being accessed strategically because it is not the easiest or quickest thing to get into and it is almost like it is a bit like a lottery if you get one that is going to help you or not*. AE cons **○** *I know it is hard to find the time during the day to complete these (KIS) but in OOH the most important things I have is background observation and base line observations.* GP **○** *In out of hours and you have a confused buddy you don’t have any background information. You have no carer to tell you why, there is no relative it is very tricky there is a good chance you are going to miss something. Then as you don’t know if they are confused normally - you don’t know anything - so that makes it tricky.* GP2
f) Record patient observations in clinical record	• In May 2016, there were a total of 731 admissions via ADOC, of which 592 were patients aged 16 or over (Table 5). Of these, 270 were for a presumed infective cause (66.2%). **Out-of-hours** **All physiological parameters present to calculate NEWS score.** • **Those with infective cause: 32 of 50 (64%)** • **Those with presumed sepsis: 10 of 29 (34%)** • **NEWS score never calculated** • **Electronic template used in 5 patients (10%)** **In-hours** **All physiological parameters present to calculate NEWS score.** • **Those with infective cause: 11 of 76 (14.5%)** • **Those with presumed sepsis: 2 of 11 (18.2%)** • Recording of observations in out-of-hours was higher than in-hours and varied between practices. Despite the out-of-hours templates being described as a less usable resource, clinicians described feeing more vulnerable in an out-of-hours setting and were more likely to record all values. Most clinicians discussed measuring and recording physiological parameters to aid diagnosis and to defend themselves if something went wrong, but were not aware if secondary care colleagues found this information useful. **○** *I feel in out of hours you don’t know the patient so well so I am very precise in out of hours of recording observations and I think it would be a good idea if more people did that*. GP • All physiological parameters were recorded less frequently for patients admitted with presumed sepsis, as opposed to an infective cause where sepsis was not suspected. One GP reported that once the decision to admit a patient had been made, further observations were not made. This was felt to be a beneficial trade-off to deal with the competing goals of efficiency versus thoroughness. **○** *I saw this man on a visit and from the moment I walked in I knew I was admitting him. We had the information that he was getting chemo and was a bit shaky. I did his temp and pulse and thought – right you are going in – so I did not do the other values.* GP2
g) Decide to admit patient	• This function was thought to vary dependent on the clinical picture and also clinician experience. **○** *I think it is variable I think it is probably clinician dependant. Experience dependant. Possibly patient dependant or practice dependant.* GP1 • The lack of time to observe the trajectory of the patient condition was reported. **○** *The fact so many other things could be going on and the rapidly changing clinical picture cause you have only 10–15 maybe 20 mins, if you are lucky, with the patients.* GP1 • Some clinicians used early warning scoring systems to aid decision making. These involve assigning a value to each physiological value and calculating a composite score to stratify risk. Others felt such scores were not helpful as routinely recording early warning scores would make normal work more difficult to do (through extra time to calculate and record scores). **○** *I do observations - I probably do a version of NEWS … and I make the clinical decision based on that.* GP2 • The overall clinical picture was felt to be a more important indicator of the severity of illness. **○** *You have got to put it together with other observations and clinical picture and the history it gives you more weight, it is all about picking up things that help you make your decision.* GP4 • *When a patient comes through SPOC we do not get KIS access – surely this could be changed.* GP1 **○** *It would be good to have access to previous notes to help decision making.* GP1
h) Transfer patient to secondary care	• One GP reported that specialty trainees, who he supervised, usually ordered an immediate ambulance if sepsis was considered whereas, if the patient was relatively stable, he may order an ambulance that would transfer the patient to hospital within one hour. Variability in this area was thought to relate to a lack of guidance on transfer urgency. **○** *I dunno…I suppose we should get a blue light ambulance .. yeah that’s what the trainees I supervise do. Sometimes I have arranged a 1 h though... I mean not if they are like very ill but if some of their obs are off but they are still well enough.* GP2
i) Communicate with secondary care	• Variability was seen in the output of this function. Secondary care clinicians reported that the number of physiological parameters communicated during admission was variable. In addition, the use of the word sepsis to alert secondary care colleagues that the patient being admitted may require immediate clinical assessment was variable. **○** *In OOH there is a variation of what information we get a lot of times .em so the girls manning the phone will still ask the same questions it just that information isn’t always to hand it is person dependent.* CAU • *So, the most important thing for us is the more warning we have - and clear communication comes is really helpful - because as soon as the word sepsis is used it will precipitate a certain response amongst our team.* AE **○** *I don’t think I have ever used the word sepsis I am admitting this patient with sepsis.* GPANP **○** *I would describe the situation rather than say sepsis maybe I should say sepsis.* GP2
j) Assess in secondary care	• It was felt that the variability of information received in admission communication and in the KIS had the potential to influence this function and result in delayed assessment, treatment and possible poorer patient outcomes. **○** *Right we know this patient is coming we are expecting him as soon as that ambulance arrives they are straight into our resus bay where the team are waiting*. CAU **○** *I think if there has been abnormal physiology it is useful to have that documented.* AE
k) Perform assessment of patient by community healthcare staff	• The output of this function was influenced by lack of available resources (thermometers, oxygen saturation monitors) and absence of controls - guidance on how to assess patients, what information should be communicated to clinical colleagues and to guide urgency of clinical review. **○** *I do not think we could record all these scores as we do not carry thermometers or sats monitors. I know that the chemo folk need admitted and we are able to call the surgery to get a GP to see them. At the weekend, we can use the SPOC [single point of contact] to directly request a GP visit but I am not sure how quickly that [visit] happens.* Community nurse
l) Make guidelines available to clinical staff	• NHS24 had electronic versions of guidelines and two GPs reported having and using an electronic smart phone application for sepsis management. Others were not aware of new guidance or did not know where it could be accessed. **○** *I have not seen the new guidelines.* GP4 **○** *I mean if there were some guidelines - get guidelines out.* GP2 **○** *I do carry the [sepsis] app.* GP1
m) Educate clinicians on sepsis management	• Educational meetings were considered valuable in raising awareness of guidelines for sepsis management by those that attended them, but many had not attended any local learning events. Other forms of delivering targeted education were suggested. **○** *Education sessions trying to get people to engage – different people like different things and meetings are not suitable for everyone so not everyone has attended before.* GP2
n) Maintain and stock equipment	• Variable access to resources such as thermometers and saturation monitors was reported by community nurses. For both in-hours and out-of-ours GPs and ANPs, this was thought to be adequate. **○** *Most of the time in ADOC you have the thermometer and stuff and have spare batteries - I have never had a problem with that.* GP1 **○** *In the surgery there is everything you need but I suppose sometimes I have to go and find stuff. I mean like a thermometer or a sats monitor.* GPANP **○** *We do not carry thermometers or sats monitors*. DNs

**Table 5:** Recording of physiological parameters admissions data

Data set	Mean age	Number of physiological parameters recorded per patient (max 6) median (interquartile range)	Temp, *n* (%)	Pulse, *n* (%)	BP, *n* (%)	Saturations, *n* (%)	Resp rate, *n* (%)	Consciousness level, *n* (%)	All physiological parameters present to calculate NEWS score, *n* (%)
Out-of-hours admissions diagnosed as possible infection (*n* = 50)	66.2	5 (1)	50 (100)	50 (100)	48 (96)	45 (90)	31 (62)	38 (76)	32(64)
Out-of-hours admissions diagnosed as sepsis or possible sepsis (*n* = 29)	66.1	5 (1)	29 (100)	28 (97)	20 (69)	26 (90)	18 (62)	22 (76)	10 (34)
In hours patients diagnosed with possible infection (*n* = 76)	Not recorded	4 (2)	53 (69.7)	66 (86.8)	40 (52.6)	53 (69.7)	42 (55.2)	37 (48.7)	11 (14.5)
In-hours patients where sepsis considered diagnosis (*n* = 11)	Not recorded	4 (1)	10 (90.9)	10 (90.9)	6 (54.5)	7 (63.6)	6 (54.5)	6 (54.5)	2 (18.2)

### Co-design of improvement intervention

Six improvement intervention themes were identified comprising of (1) feedback to facilitate reflective learning, (2) improving communication pathways, (3) use of early warning scores, (4) improving electronic template for recording physiological parameters, (5) provision of sepsis education and (6) improving KIS completion.

1. Feedback to facilitate reflective learning

Many of the professionals interviewed stated that they wanted feedback on their own practice to facilitate learning but this was rarely given. A system-based reflective tool was developed to direct practice teams to reflect on their current systems. This could be used to investigate events when patients were diagnosed with sepsis or to prospectively examine their systems and share learning within teams on how they manage difficult system conditions. The tool provided data from the FRAM to encourage individual and team reflection on their role in the overall system and how this influences other parts of the system. This included how work-as-imagined and work-as-done differ in areas such as arranging clinical review, assessing patients and communication across interfaces.

**Fig. 2:**
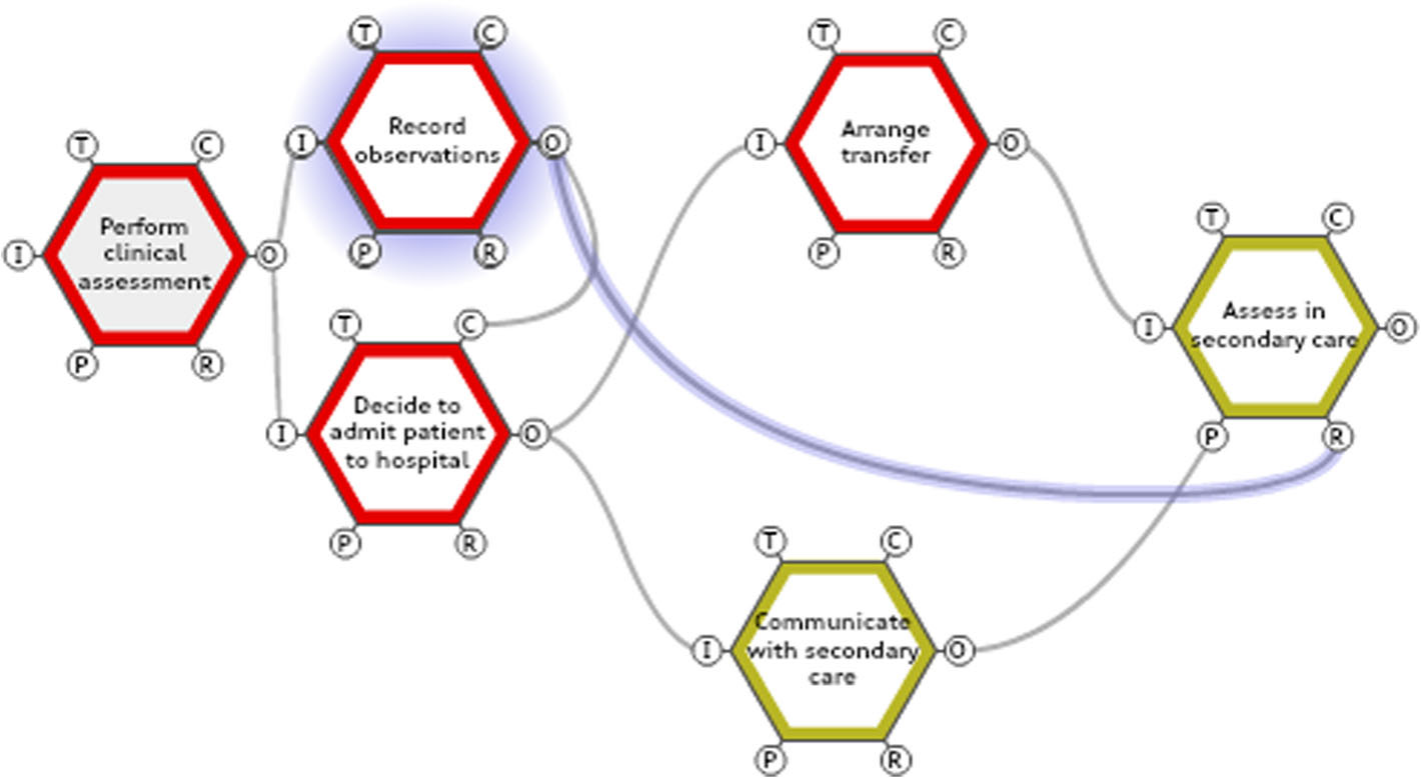
Extract from Functional Resonance Analysis Method (FRAM) model demonstrating importance of recording observations to other functions in the system

For example, practice teams were encouraged to analyse their own recording of physiological parameters and compare this to the data collected when constructing the FRAM. It was felt that recording, interpreting and communicating the individual physiological parameters was essential to successfully recognise and manage patients who may be at risk of sepsis. This is demonstrated in the FRAM model which shows that the function <record observations> links to four other functions (<decide to admit patient>, <communicate with secondary care>, <transfer patient to secondary care> and <assess in secondary care>). Variability in this function could influence all of these functions (Fig. 2).

Clinicians were much more likely to record physiological parameters in an out-of-hours setting than an in-hours setting. This was due to feeling that out-of-hours work was riskier as they did not know the patients as well as those seen in their own practices during normal in-hours working.

I feel in out of hours you don’t know the patient so well so I am very precise in out of hours of recording observations and I think it would be a good idea if more people did that. GP1

When patients were admitted and the diagnosis was thought to be sepsis, it was less likely that all physiological parameters were recorded. Clinicians recognised that this was due to employing an efficiency thoroughness trade off based on making a rapid decision to quickly admit patients who appeared acutely ill and so did not record all parameters.

I saw this man on a visit and from the moment I walked in I knew I was admitting him. We had the information that he was getting chemo and was a bit shaky. I did his temp and pulse and thought – right you’re going in – so I didn’t do the other values. GP2

Although this is an effective trade-off from the GP perspective, this physiological information is considered extremely important when the patient is assessed in secondary care which was not fully appreciated by those in the community.

I think if there has been abnormal physiology it is useful to have that documented. AE

Teams were asked to reflect on their own data and the presented data to consider if changes to local systems were required. Trade-offs and performance variability are needed in complex healthcare systems, but it is essential that we understand the potential effects at a local and wider system level through exploring and understanding the system [34, 46].

2) Communication pathways

Physiological parameter values were important when the patient is assessed in hospital (Fig. 2). The results of this project fed into existing work-streams on communication between primary and secondary care. During telephone admission calls to the secondary care combined assessment unit, all physiological parameters will routinely be requested by receiving staff. This allows a degree of flexibility for community staff while still encouraging communication of all parameters.

3) Use of early warning scores

Although early warning scores have been endorsed as a way to detect acute illness due to sepsis, there were mixed opinions on the use of early warning score.

There is much more of a push to do observations which I think gives you more of an objective measurement which might push someone towards a potential sepsis rather than just an unwell diagnosis and make you act a bit more promptly. GPST3

I think [a score] gives you more weight to make the decision that this person is unwell - Even young people for example could be septic and still look alright you know. GP4

I don’t think it would change what I do much it would just be more to stimulate me to remember more things. GP2

Yeah and I think a lot of the times when you have this scoring system we are taking away people’s common sense it is just a scoring system, it’s just a helpful tool it shouldn’t replace your clinical judgement. CAU senior nurse

There is less evidence for the use of a “one off” early warning score in the community to identify patients with possible sepsis as opposed to repeatedly recording early warning scores to identify clinical deterioration of a patient. It was felt that the use of an early warning score did not fit with the way that GPs currently worked as they were more likely to consider the whole clinical situation. They felt that the interpretation of parameters and the communication of concern between health professionals were more important than the calculation of the score which also increased workload.

You have got to put it together with other observations and clinical picture and the history it gives you more weight, it is all about picking up things that help you make your decision. GP4

There was concern by some clinicians that if early warning scores were used as part of a QI intervention, compliance would be rigidly monitored reducing scope for clinicians to adapt their behaviour to suit the patient in front of them and the work conditions experienced. Instead, a less rigid approach was recommended focussing on the social aspects of communicating across interfaces and providing opportunity for feedback to encourage reflection on when and why to record physiological parameters.

But people want every box ticked. Because someone will audit it, someone will look at it and then they will come round and go like we have had a complaint from a patient who had a sore throat turned out two days later he had quinsy you don’t seem to have recorded saturations on him. GP1

**Fig. 3:**
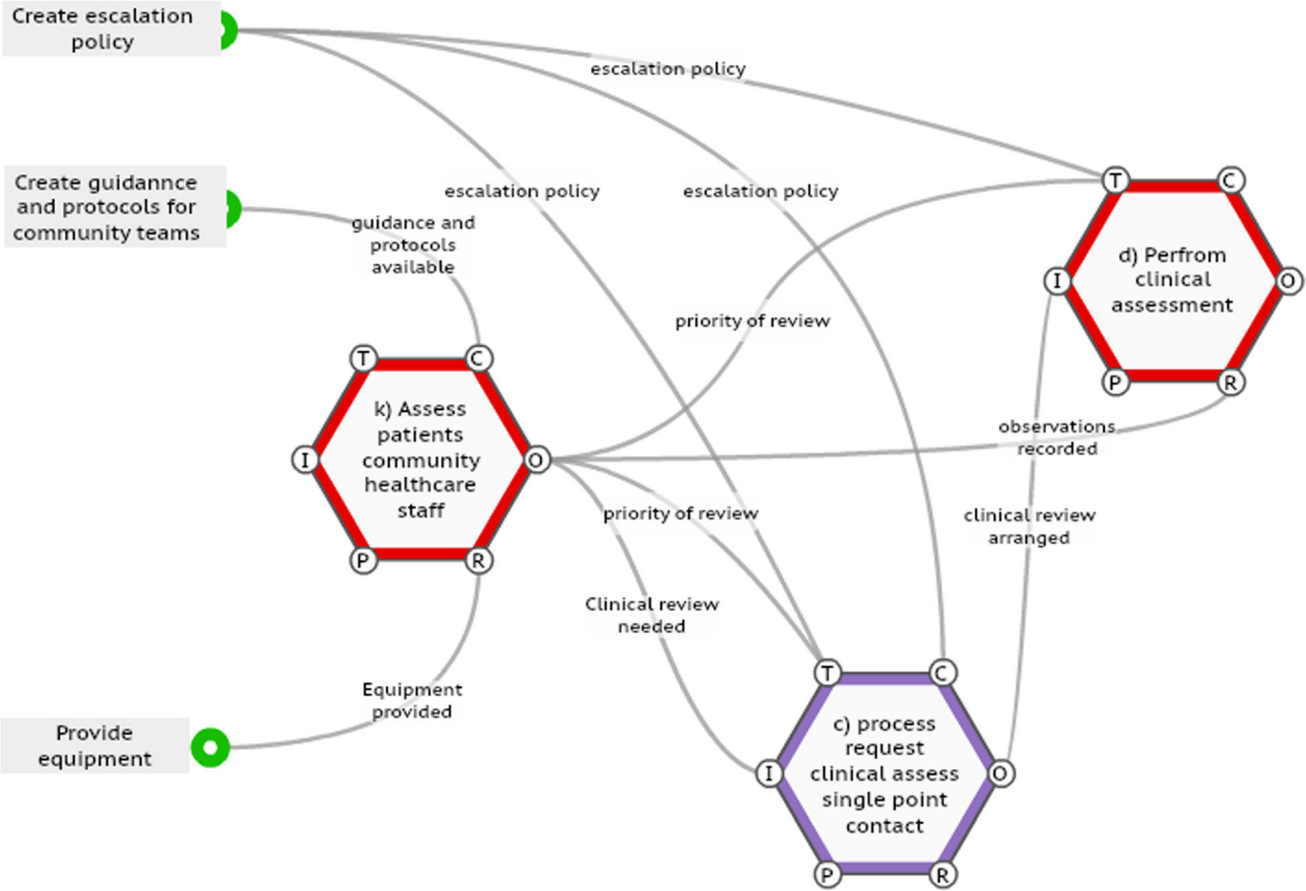
Extract from Functional Resonance Analysis Method (FRAM) model demonstrating extra functions (on left) that will be needed if system is changed

Despite this, it was agreed that the early warning score may be useful to communicate with professionals in other parts of the system, for example, ambulance services or community nurses. To test this, a pilot project was planned involving community nurses using early warning scores to assess patients and communicate with clinicians in an out of hours setting. Study of the FRAM allowed anticipation of potential problems when implementing these changes by identifying functions that would be influenced by the intervention (Fig. 3). Systems need to be in place to ensure availability of resources such as thermometers and oxygen saturation monitors for community nurses. The output of community nurse assessment will direct the priority of clinical review required. Communication and escalation policies will be required to direct this process for the single point of contact and clinicians.

4) Electronic template for recording physiological parameters

The existing electronic templates were non-intuitive and did not fit with the way work was currently done. Because of this mismatch, clinicians used workarounds such as hand-writing values or typing them into the electronic record as free text. The template available on the in-hours system was considered more useful as it provided information to aid interpretation of results but it often still took time to find and open. Some practices had created shortcuts to allow its use within the consultation—a code, that when typed, automatically opened the template. The out-of-hours template was rarely used as values had to be entered after the clinician had left the patient and so any guidance from the template came too late.

The out-of-hours template makes it more difficult – you see it when you are back in the car writing up the case after you have made your decision – it’s too late. I think if it was quick, easy and straightforward you might get better recording (of observations). GP2

The stakeholder group recommended the design of an electronic template that fits with the current work to make its use as simple as hand written notes or free text entries. Work is underway to develop a template to alert clinicians in real time to abnormal physiological parameters that may prompt recording of all relevant parameters with automatic calculation of an early warning score.

5) Provision of sepsis training

By exploring multiple perspectives, the FRAM helped identify the conditions of work that result in divergence of work-as-imagined by clinicians and work-as-done by administrative staff. Clinicians generally thought that their administrative staff could accurately identify patients who may need early assessment and knew how to arrange this. However, administrative staff felt that they had no training or guidance on how to identify patients who may be at risk of sepsis and often had no clear advice on how to arrange rapid review.

In general, our staff are good at saying this person doesn’t sound well and they are concerned and they don’t call often and they let us know so they will put it onto the emergency doctor. GP3

I don’t know if I would necessarily recognise it in a patient coming in because a lot of it is like fever and sickness - it could be anything. Training or a checklist may help. Receptionist 2

System conditions affected the output of the function describing staff arranging clinical review and so, even with training, staff may not be able to successfully identify and deal with patients who may have sepsis. This information was used to design educational materials that accompany the system-based reflective tool. The aim is to allow teams to consider how the sepsis education material can be applied in their own setting to improve care. For example, if staff are more aware of the vague symptoms that may indicate risk of sepsis (such as confusion) they need a way to raise their concerns with clinical staff and the clinical staff need a way to respond flexibility dependent on the situation (such as knowledge of patient and competing priorities).

It can be quite hard on a Monday morning when you have got lots of patients waiting for an on-the-day appointment and we just get a sea of people it would be quite hard to say then could you give me indication of the problem. Receptionist 2

I think it is easy for us to recognise someone that comes in with chest pains rather than someone who comes in with sepsis. Receptionist 1

I need to be able to go to someone comfortably and say I am just raising this. To make you aware as I am concerned. Receptionist 2

6) KIS completion

**Fig. 4:**
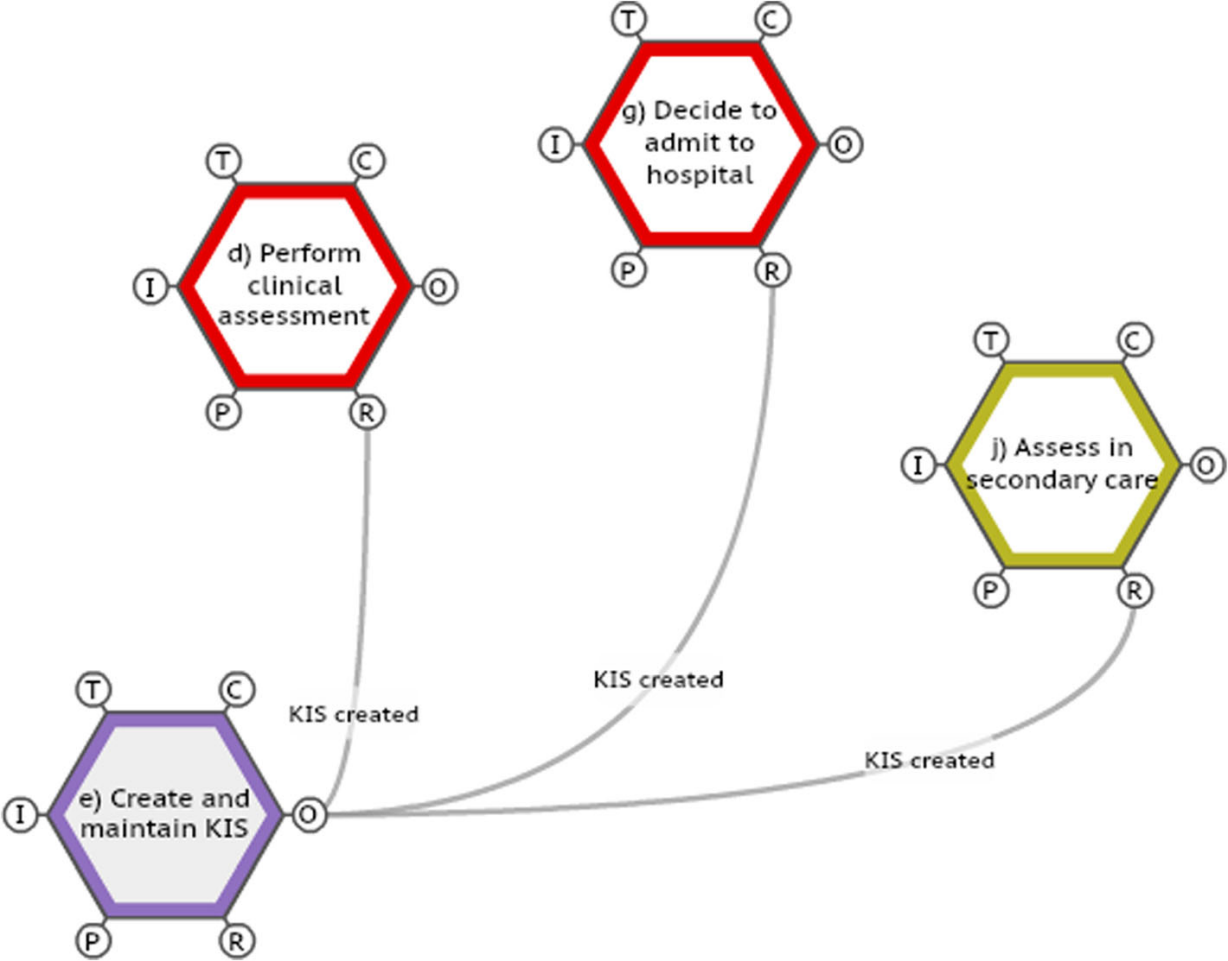
Extract from Functional Resonance Analysis Method (FRAM) model demonstrating the importance of the Key Information Summary (KIS) to several functions in the system

The importance of the Key Information Summary became clear when interviewing professionals in different parts of the system and was demonstrated within the FRAM model (Fig. 4). Work was already underway locally to improve KIS completion in terms of identifying patients appropriate for KIS completion and recording relevant details such as usual oxygen level, pulse, blood pressure, level of confusion and wishes regarding ceilings of care. The FRAM model was used to inform further work in this work-stream as well as providing evidence in the system-based reflective tool of the importance of this task elsewhere in the system.

I think it is variable sometimes it is excellent (the KIS) and it makes such a difference - and then other times it isn’t - and I think that is probably one of the reasons why it is not being accessed strategically because it is not the easiest or quickest thing to get into and it is almost like it is a bit like a lottery if you get one that is going to help you or not. AE consultant

I know it is hard to find the time during the day to complete these (KIS) but in OOH the most important things I have is background observation and base line observations. GP

It was also identified that the KIS was not available when the SPOC was used to refer patients to primary care out-of-hours clinicians. Information Technology systems were altered to solve this problem.

**Fig. 5:**
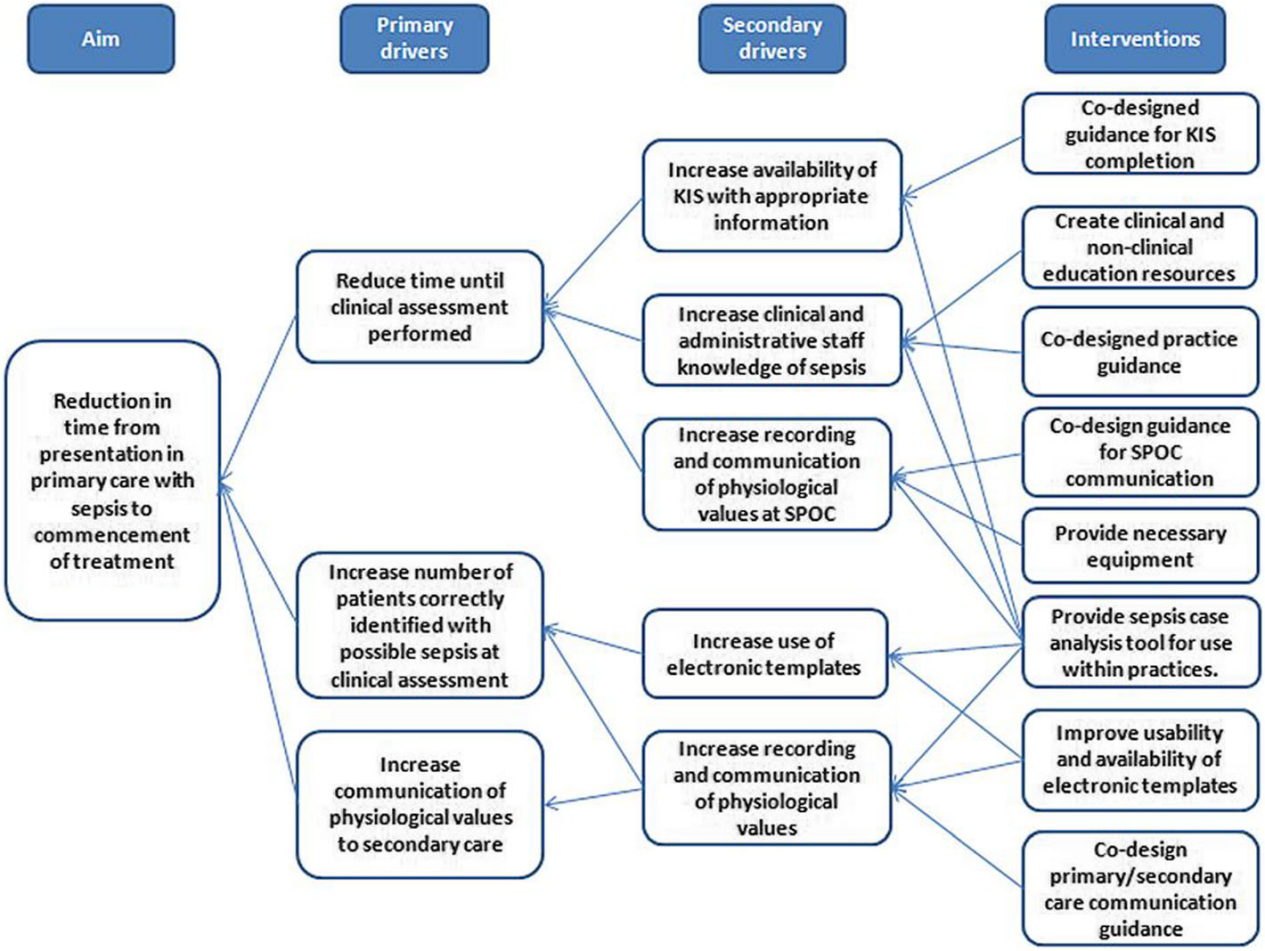
Preliminary driver diagram of improvement intervention for management of sepsis in primary care

Following consideration of each improvement theme, consensus was reached on the design of a Driver Diagram and multi-component improvement intervention (Figure 5, Appendices 1 and 2). It was agreed that the overall purpose of the system was the identification and management of sepsis in the community. The boundary of the system for improvement excluded NHS24 as this was a national organisation over which we would have little influence.

## Discussion

In this paper, we described how a FRAM model of the complex system to identify and manage sepsis in primary care was constructed to understand how conditions of work and system interactions influenced everyday work in a regional NHS Board. This information directly allowed reconciliation between improvement suggestions from frontline staff and current works systems and informed the design of a multi-component improvement intervention to improve overall system functioning.

Despite the complex systems that exist in healthcare, many improvement projects fail to take a “systems approach”, or misunderstand and misapply this concept. Many seek to introduce new procedures in a top-down manner or implement change and improvement at the level of individual performance through, for example, audit and feedback strategies [24, 47]. As a result, the focus of many interventions has been on single-system components such as performing a clinical assessment more reliably or effectively [48–51]. Improvement interventions often target the person through education and training, protocol dissemination or recommend the use of a tool or technology, such as an IT template or early warning scores [49–51]. Educational interventions alone are considered weak as they depend on memory of training whereas introducing tools or technology to aid recall is considered to be of intermediate strength as an improvement intervention [52]. Evaluation of such interventions involves measuring compliance (of the component targeted) with the proposed change. It is thought that this attempt to reduce process variation will improve health outcomes [53]. However, the evidence frequently demonstrates that these types of interventions often fail to have the sustainable impact anticipated leading to missed opportunities to improve system performance and reduce avoidable patient harm [28].

Rather than persisting with linear, cause and effect approaches, the use of a complex system lens may help to maximise the impact of improvement interventions [26, 27]. One way to do this is to engage the people in the system who are expert at doing the work to both understand the system and identify potential improvements [26]. In this way, improvement strategies can be co-designed that consider important contextual factors when implementing change and include strategies to support local adaptation to cope with the conditions faced [34, 54]. In this study, the interventions did not over specify work by mandating and measuring the use of early warning scores but encouraged recording and communication of physiological parameters while allowing clinicians to adapt if needed based on the conditions they experience. The edges of systems are blurry and interact with other systems [26]. As such, treating sepsis identification as a standalone system, and educating administrative staff on its identification, is unlikely to be effective unless consideration is given to the other task they are doing and the other systems with which they are interacting. We believe that the method described in this study is one way to involve multiple perspectives in the co-design of change and will add value to existing quality improvement methods.

It may be argued that simply discussing implementation of the improvement suggestions with a multidisciplinary team would yield similar results. The benefit of using the FRAM is that it allowed the qualitative and quantitative data to be synthesised and the whole system to be conceptualised. By identifying the conditions and interactions that influence work and cause variable function output, we believe it helped support clinical teams to consider where improvement efforts should be targeted. Constructing the FRAM model is a trade-off between showing all related functions and ensuring that it is useable and understandable. It may be argued that the FRAM could describe many other background functions (such as <manage staff capacity>) and links to other systems (such as <patient obtain access to laboratory results>). FRAM models can be constructed with different levels of resolution. For example, if the function <process request for clinical assessment – GP surgery> was the main object of improvement, this could be broken down to include all the functions needed to complete this task, such as <answer the telephone>. This has potential to increase the complexity of the FRAM model by identifying more interrelated functions. The level of detail required is dependent on the data collected and validated by those doing the work. If links to other systems significantly influence work in the system under study, then they should be included, and if variability in a specific task within a function (such as how the telephone is answered) is important, then it should be included as a separate function [36].

Consensus already exists on how improvement interventions should be described and reported [55, 56] and recent recommendations to improve the design of improvement interventions in complex systems have been published [23]. These include rigorously defining the problem, co-designing improvement interventions, use of a programme theory and considering the interaction between the social and the technical aspects of change. We have described one way to rigorously explore and understand the system to identify potential problems by exploring local work-as-done by frontline staff—for example, expected actions of administrative staff when patients present with possible sepsis and the lack of community nursing equipment. Improvement ideas were generated and interventions co-designed with frontline staff. The reflective sepsis tool promoted co-design of specific practice level interventions. It may be argued that this will produce a new work-as-imagined from which people will have to vary when conditions change in an unexpected way. However, the tool encourages repeated team reflection on performance to understand different perspectives on how the system functions and will support further adaptation to guidance to bring work-as-imagined and work-as-done closer.

The FRAM explored how the system worked and how interactions, resources, controls and time influence output. This allowed us to develop a programme theory, presented in the Driver Diagram (Fig. 2), that defines how interventions may lead to overall system improvement and how each intervention could be evaluated [57]. This will be used by local teams to learn about and adapt local processes to maximise success and is currently being piloted. As recently recommended for improvement interventions in complex systems, we have agreed a measurement of the final outcome of interest allowing for local adaptation of processes to create success [46].

The participatory approach we adopted helped us to explore the social and technical aspects of change. Increasingly, the use of risk stratification and early warning scores are being promoted in primary care but there is little evidence of their benefit as part of a one-off pre-hospital clinical assessment [9, 10]. The key stakeholder group felt that the social “processes” that lead to the interpretation and communication of the output of these tools (the actual physiological parameters and an indication of clinical condition) are what will ultimately influence the quality and safety of care [58].

Many factors that should be considered to maximise implementation and sustainability of improvement interventions within complex system have been described [59]. These include how the intervention fits with current work, demonstrating the benefits of the intervention and the ability to adapt it to local conditions [59]. Considering these factors can help understand why measuring the use of early warning scores as a quality improvement process measure was rejected by the key stakeholder group. The current electronic templates are not simple to use and do not fit with the way work is currently done. The benefits were not obvious to community clinicians—although there may be benefits in other parts of the system. There was also concern that if they were used as part of a QI intervention, compliance would be rigidly monitored reducing scope for clinicians to adapt their behaviour to suit the patient in front of them and the work conditions experienced. Instead, a less rigid approach was recommended focussing on the social aspects of communicating across interfaces and providing opportunity for feedback to encourage reflection on when and why to record physiological parameters.

This study has several limitations. First, several key stakeholders were not involved—most notably patients, home care teams and the Scottish Ambulance Service. We did not know if this approach would work and wished to initially test it with healthcare professionals. Better integrated patient participation will be sought to develop the improvement intervention design. The study included small numbers of participants in each professional group. This did not present a problem in the construction of the FRAM model and it appeared that data saturation was achieved for improvement suggestions. However, with more participants, it is possible other ideas for change may have been generated. The FRAM model was constructed based on work-as-disclosed by participants and observation of actual work may have revealed other ways of working. Interviewees may have been guarded in their description of how they completed work as they were speaking to a local GP; however, this made access to participants’ easier and improved understanding of contextual factors such as the limitations of existing electronic templates. Transcripts were not returned to participants for checking. Data from NHS24 only included patients who received an out-of-hours clinician review, and did not include how often an emergency ambulance was called. It may be that NHS24 identify most patients with sepsis and arrange ambulance transport. Nevertheless, it allowed assessment of the variability of output of the function of arranging clinical review that may delay transfer to hospital. Similarly, the low rate of GP practice participation in data collection may mean levels of recording are not representative but they do demonstrate variability which was the main objective. The stakeholder meeting held to agree the improvement intervention did not include representation from all staff groups but their perspective was considered through the discussion of the suggested improvement interventions. The methods used to explore and understand the system require considerable experience and time investment that will not be available in all improvement projects. FRAM model construction through facilitated group discussion is successfully used elsewhere and this may be a more time efficient method to allow wider application and inclusion of more participants from each professional group [40, 41]. This method has only been used to design the intervention, and future evaluation of the intervention is required. Similarly, the method has only been tested in a single regional health board and further evaluation of its application in different settings is required. A full evaluation of the impact of this approach is planned and further research on the application of this method in different healthcare areas is required.

## Conclusion

We have demonstrated the use of FRAM in a complex system to aid the design of a quality improvement intervention for identifying and managing sepsis in a single regional NHS board. This allowed an exploration of how conditions and interactions influence performance and output and how improvement suggestions from frontline staff could be reconciled with current work systems.

### Additional file

Additional file 1: Consolidated criteria for reporting qualitative studies (COREQ): 32-item checklist. (DOCX 16 kb)

## Figures and Tables

**Fig. 6:**
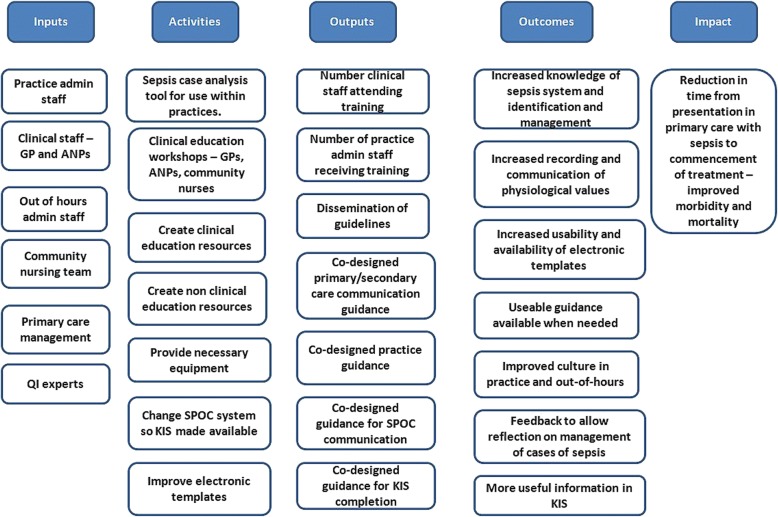
Sepsis improvement intervention logic model

## Appendix 1

**Table 6:** System improvement intervention informed by SEIPS 2.0 model [60]

Part of work system	Improvement aim	How will this be done?	Anticipated outcomes	Evaluation
Person	1. Increase administrative staff knowledge on sepsis. 2. Increase clinical staff knowledge on the identification and management of sepsis in the community.	1. Development of sepsis case analysis tool for use within practices. 2. Education session for receptionist staff, production of learning pack deliverable in practices. 3. Clinical educational sessions and production of accessible educational material (e.g. webinar, online module, dissemination of learning pack) Containing summary of guidelines, systems approach, recommendations and their rationale for standardising communication to increase recording of physiological parameters. 4. Training for adult community nurses on sepsis management and measuring and interpreting physiological values.	1. Reception staff aware of how sepsis will present and possible red flags—prompting them to arrange sooner clinical review. 2. Increased knowledge of guidelines, available tools (IT templates and NEWS), appreciation by clinical staff of reasons for recording and communicating values and how this can be achieved. 3. Earlier recognition by community adult nurses of septic patients and more effective communication of concern resulting in sooner clinical review by GP.	1. Evaluate satisfaction with training and other educational materials. 2. Evaluate change in attitude and knowledge following training.
Tools and Technology	1. Provide adult community nurses with required resources—thermometers and saturation monitors. 2. Facilitate recording of physiological values.	1. Resources provided through health board funding. 2. Improved IT systems that are a useful resource, available when needed that may help positively constrain behaviour. 3. Dissemination of existing in-hours IT templates to practice managers with instructions on how to use short cuts to open—work with frontline staff to improve out-of-hours IT systems	1. All necessary equipment available. 2. Easier to record physiological values. 3. Awareness of guideline that supports everyday work (positive control). Patients who are potentially septic are identified earlier.	1. Assess via survey—satisfaction with created protocols and templates. 2. Survey staff to determine if protocols and templates are a beneficial control and represent work-as-done. 3. Measure use of templates.
Tasks	1. Increase recording and communication of physiological parameters 2. Improve the ability of practice administrative staff to identify patients who may have sepsis. 3. Improve completion of Key Information Summary to include—the recording of risk factors for sepsis and normal physiological parameters	1. Development of sepsis case analysis tool for use within practices. 2. Through educational events that describe importance of recording values in other parts of the system. 3. Co-design guidance with community nurses following education on sepsis—potentially positive control. 4. Co-design protocol for communication between primary/secondary care. 5. Co-design guidance with practice administrative staff following educational sessions—potentially positive control. 6. Improvement in KIS completion will be achieved through existing programme of work—sepsis work will feed into this.	1. Increase recording and communication of physiological parameters. 2. More accurate and useful information contained in KIS—allows interpretation of physiology to facilitate accurate diagnosis in out-of-hours and secondary care.	1. Measure use of protocols and templates to determine if they represent work-as-done. 2. Evaluate information contained in KIS—through existing GP cluster and locality work. 3. Evaluate patients admitted with sepsis to determine if all parameters recorded—results to be used for reflection and not as a performance indicator.
Internal Environment	1. Develop practice culture where receptionists can interrupt clinicians if needed. 2. Improve culture within out-of-hours to reduce concern regarding auditing of data.	1. Development of sepsis case analysis tool for use within practices. 2. Co-design of protocols with clinical and administrative staff in practices following reception and GP training. 3. Regular reinforcement of use of data and incident investigation for learning—recording of observations or early warning scores should not be used as a performance indicator without appreciation of the context within which the patient was assessed.	1. Receptionists know when to adapt behaviour—when to seek early review and have confidence to implement—supports staff wellbeing and improves performance. 2. Feedback from incident investigation and data used for learning—supports staff wellbeing.	1. Survey of perceptions of culture.
Organisation	1. KIS available when SPOC used—resource provision. 2. Improve communication when out-of-hours community healthcare staff use the single point of contact. 3. Improve communication between primary/secondary care	1. Change system to ensure KIS available—arranged with out-of-hours leaders. 2. Following education sessions with adult community nurses and out-of-hours administrative staff—co-design guidance for communication including communication of physiological values. Potentially positive behaviour control. 3. The sepsis work would feed into existing cross interface programme boards—co-design protocol for communication between primary/secondary care.	1. Normal values available for out-of-hours and secondary care clinicians to facilitate early diagnosis and treatment. 2. Awareness of guideline that helps work (positive control). Patients who are potentially septic are identified earlier.	1. Evaluation as above.
External Influences	1. Sepsis management prioritised by Health board. 2. Nice Guidelines widely distributed 3. Reflection on management of sepsis patients with other GP practices	1. Report sent to health board for discussion and approval at Primary Care Leadership committee. 2. Dissemination guidelines and sepsis app as part of educational intervention-—potentially positive behaviour control.	1. Resources available to implement and evaluate changes. 2. More patients managed following guidance.	1. Use of guidance can be evaluated following educational events using a survey.
Processes	1. Increased rates of provision of relevant physiological values when admission arranged by primary care clinicians. 2. Increased rates of provision of relevant physiological values when community healthcare staff contacts out-of-hours services.	1. Work with secondary care sepsis leads—for all admissions receiving team will request all physiological parameters—GP expected to provide values when relevant—educational sessions detail when it is relevant. This will include all admissions with infective, cardiac or respiratory cause. Efficiency thoroughness trade-offs may lead to performance variability and this should be recognised. 2. SPOC will use a template and ask for all physiological parameters.	1. Improved communication of physiological values so secondary care aware of admissions and have values from community for comparison. Results in quicker assessment and initiation of appropriate treatment. 2. Out-of-hours staff will be aware of severity of illness of patient and, if necessary see sooner and ensure treatment initiated sooner, resulting in improved healthcare outcomes.	1. Measure rates of communication of relevant values when SPOC used and at admission. 2. Survey—perceptions of clinical staff in acute care hub to new system for adult community nurses.
Outcomes	1. Reduce time from contacting health services to receiving antibiotics for ten patients with a confirmed admission diagnosis of sepsis per month.	1. Long term outcome of all above measures	1. Improved mortality and morbidity outcomes for patients presenting to primary care with sepsis.	1. Measure for ten patients per month and feedback to all GPs and ANPs. Once baseline measure obtained specific target will be set.

## Abbreviations

ADOC: Ayrshire Doctors on Call
ANP: Advanced nurse practitioner
CMAU: Combined Medical Assessment Unit
COREQ: Consolidated criteria for reporting qualitative research
ED: Emergency department
FMV: FRAM Model Visualiser
FRAM: Functional Resonance Analysis Method
GP: General practitioner
GPST3: General Practitioner Specialty Trainee 3
KIS: Key Information Summary
NHSAA: National Health Service Ayrshire and Arran
QI: Quality improvement
SPOC: Single point of contact
WAD: Work-as-done
WAI: Work-as-imagined
